# A Brief Mobile-Augmented Suicide Prevention Intervention for People With Psychotic Disorders in Transition From Acute to Ongoing Care: Protocol for a Pilot Trial

**DOI:** 10.2196/14378

**Published:** 2021-02-08

**Authors:** Colin Depp, Blaire Ehret, Jennifer Villa, Dimitri Perivoliotis, Eric Granholm

**Affiliations:** 1 Department of Psychiatry University of California, San Diego La Jolla, CA United States; 2 VA San Diego Healthcare System San Diego, CA United States; 3 Department of Psychology San Diego State University San Diego, CA United States

**Keywords:** prevention, mental health services, psychosis, technology

## Abstract

**Background:**

People with serious mental illnesses (SMIs) are at exceptionally high risk for lifetime suicidal ideation and behavior compared with the general population. The transition period between urgent evaluation and ongoing care could provide an important setting for brief suicide-specific interventions for SMIs. To address this concern, this trial, SafeTy and Recovery Therapy (START), involves a brief suicide-specific cognitive behavioral intervention for SMIs that is augmented with mobile phone interactions.

**Objective:**

The primary aim of this pilot trial is to evaluate the feasibility, acceptability, and preliminary effectiveness of the intervention.

**Methods:**

A 6-month pilot trial with 70 participants with a diagnosis of bipolar disorder, schizophrenia or schizoaffective disorder, and current active suicidal ideation were randomized to START or START with mobile augmentation. START consists of 4 weekly sessions addressing early warning signs and triggers, symptoms influencing suicidal thinking, and social relationships. Recovery planning is followed by biweekly telephone coaching. START with mobile augmentation includes personalized automated cognitive behavioral therapy scripts that build from in-person content. Participants were evaluated at baseline, 4 weeks (end of in-person sessions), 12 weeks (end of telephone coaching), and 24 weeks. In addition to providing point estimates of feasibility and acceptability, the primary outcome of the trial was the change in severity of suicidal ideation as measured with the Scale for Suicide Ideation (SSI) and secondary outcome included the rate of outpatient engagement.

**Results:**

The trial is ongoing. Feasibility and acceptability across conditions will be assessed using t tests or Mann-Whitney tests or chi-square tests. The reduction of SSI over time will be assessed using hierarchical linear models.

**Conclusions:**

The design considerations and results of this trial may be informative for adapted suicide prevention in psychotic disorders in applied community settings.

**Trial Registration:**

ClinicalTrials.gov NCT03198364; http://clinicaltrials.gov/ct2/show/NCT03198364

**International Registered Report Identifier (IRRID):**

DERR1-10.2196/14378

## Introduction

### Suicide in Serious Mental Illnesses

The burden of suicide is exceptionally high in serious mental illnesses (SMIs) such as bipolar disorder and schizophrenia. The lifetime risk of suicide in people with SMIs is 5% to 10% [1,2], which is 12 times the rate in the general population [3]. Recent evidence indicates that direct interventions (ie, those that specifically target suicidal thoughts, such as cognitive behavioral therapy [CBT] techniques) are more effective in reducing suicide risk than indirect interventions (eg, those that target depressive symptoms and promote treatment engagement) [4,5]. Unfortunately, most clinical trials of direct interventions exclude patients with psychotic symptoms or disorders [4,6]. Only a handful of trials of psychosocial interventions have been evaluated for their impact on suicidal ideation or behavior in schizophrenia (some of which are suicide-specific CBT [7,8]), and data on psychosocial interventions in suicide prevention are *virtually nonexistent* in bipolar disorder [9].

### Intervention Targets for Suicide Prevention in People With SMIs

To address these gaps, the 2 key questions are as follows: (1) How might the content of suicide prevention interventions be adapted for people with SMIs? (2) Where and when would such interventions fit within the care continuum? One adaptation consideration is that people with SMIs appear to have some unique characteristics of suicidal ideation and behavior as well as the transition between them. For example, in psychotic disorders, suicidal ideation may be intertwined with hallucinations, suicidal ideation appears to be less transient [10], and suicidal ideation is more likely to be associated with suicide attempts than in people without psychosis [11]. The means used to attempt suicide are also different, and a history of psychosis is overrepresented in those with severe attempts [12]. Moreover, social support is frequently limited in SMIs [13], and people with SMIs may be less likely to self-initiate the use of suicide prevention services such as crisis lines [14]. Finally, although psychotherapeutic interventions such as CBT are effective for SMIs, they are typically adapted to accommodate aspects of these illnesses that may interfere with skill acquisition, such as cognitive impairments [15,16].

There are also unique considerations for fitting suicide prevention interventions into the care continuum in SMIs. A higher proportion of service use is in acute and outpatient specialty mental health services compared with primary care. Furthermore, services tailored to people with SMIs are increasingly tailored to reduce barriers to access given the high rates of disengagement from care. One emerging site of potential deployment for brief suicide prevention interventions for people with SMI is walk-in or *same-day* clinics. Walk-in clinics provide access to immediate unscheduled psychiatric evaluation and are the best practice in both the Zero Suicide framework [17] and for service engagement of people with SMIs [18]. These clinics aim to increase access to mental health care and provide an access point to initiate ongoing psychiatric outpatient care [19,20]. However, only a minority of patients seen in urgent care for suicidal ideation actually go on to engage in follow-up outpatient care [21].

### SafeTy and Recovery Therapy

To address the gaps described earlier, we developed a brief suicide-specific intervention adapted for SMIs called SafeTy and Recovery Therapy (START). The intervention builds from prior work in CBT for suicide and SMIs. START consists of 4 sessions intended to fit within a typical gap period between urgent and ongoing care and to successively build suicide-specific skills. Furthermore, this brief in-person individual psychotherapeutic intervention is integrated with an automated mobile intervention. Emerging research has examined mobile health and telemonitoring interventions in suicide prevention [22-24], although less so in SMIs. Ecological momentary interventions (EMIs) couple brief in-person CBT with automated mobile assessment linked with personalized intervention content in SMIs [25-28] and deliver automated therapeutic content that extends the content of in-person CBT to everyday life. The role of EMIs in START is to promote recall and engagement in personalized, adaptive thoughts and behaviors aimed at suicide prevention by employing content collaboratively developed during in-person sessions.

To evaluate the START intervention, we developed a pilot clinical trial using a deployment-focused approach. The goals of this pilot trial were to evaluate the feasibility, acceptability, and preliminary effectiveness of the intervention. Here, we report our study design, intervention approach, and related considerations, which we hope will be informative for research on suicide-specific interventions for SMIs. Of particular interest, may be, design decisions in suicide-specific intervention clinical trials on SMIs, informed by guidance from the National Institute of Mental Health [29]. This study has the following aims:

- Aim 1: to refine intervention content and safety protocol with input from community stakeholders.
- Aim 2: to evaluate feasibility, engagement, impact, and preliminary comparison of START with Mobile Augmentation versus START alone.

## Methods

### Study Deployment Planning

This study is deployed in the public mental health system in San Diego, California. Study deployment relied on collaborative meetings with leadership, triage, and outpatient clinicians. Key components of these meetings included (1) a review of the draft manuals, study materials, and mobile app; (2) specifications of research team community communication plans, roles, documentation, and reporting protocols; and (3) emergency and safety planning for participants, research staff, and clinicians delivering the intervention. Front-line staff were afforded the opportunity to suggest and improve the approach, for example, where same-day clinics were in separate locations from outpatient centers, a suggestion was to split the sessions across these sites to allow participants the opportunity to transition sites and become comfortable with the outpatient clinic. To reduce the burden on sites and to increase the likelihood of consistent appropriate referrals, we held additional meetings with triage providers during in-services and created a *pocket guide* detailing the study inclusion or exclusion criteria and procedures.

### Recruitment Sites and Screening

Usual care in walk-in settings involves a diagnostic interview or intake with a triage provider (typically a social worker) and a psychiatric evaluation, which includes a standardized screening for suicidal thoughts and behavior. The outcome of these evaluations may include acute stabilization or hospitalization but is most frequently the initiation of medication treatment, linkage to resources, and an outpatient follow-up appointment for psychosocial care. The focal population of these clinics are people with SMIs, and individuals who do not meet the criteria for SMIs are referred to as primary care. To fit within these high acuity settings, our research screening is kept minimal and includes only the basic eligibility criteria, diagnostic screen, and recent and lifetime suicide history screening with the Columbia Suicide Severity Rating Scale (CSSR-S), lifetime version [30].

### Eligibility

Participant inclusion criteria are as follows: (1) aged 18-65 years, (2) *Diagnostic and Statistical Manual of Mental Disorders, Fifth Edition* diagnoses of bipolar disorder, schizoaffective disorder, or schizophrenia (confirmed by the Mini International Neuropsychiatric Interview [31]), and (3) suicidal ideation, defined as CSSR-S≥2 in the past 1 month and/or a suicide attempt in the past 3 months as identified by the CSSR-S. Additional inclusion criteria are as follows: (1) plans to remain in the region for ≥6 months and pending appointment for outpatient mental health treatment initiation and (2) capable of informed consent. Exclusion criteria are as follows: (1) not English speaking, (2) inability to complete the assessment battery, (3) insufficient visual acuity or manual dexterity to navigate a touch screen, (4) current intoxication requiring immediate detoxification or an outpatient plan directed to substance use disorder (not mental health) services, and (5) under conservatorship requiring proxy consent. Given that the population is vulnerable to impaired decisional capacity, we confirm the capacity to consent with a brief measure [32].

A key consideration for inclusion criteria was the floor and ceiling for suicide risk. We selected a floor for our suicidal ideation measure at active thoughts or higher (≥2 on CSSR-S), consistent with a recent clinical trial [33]. As our study is embedded in an urgent screening setting, triage provides a *ceiling* which is voluntary or involuntary hospitalization on the day of evaluation. Finally, due to high base rates in the population, we included people with active substance use disorder, provided that they do not require acute detoxification as a next step following triage, and we also included those with unstable housing.

### Therapist Training

Therapists for the study are employees of the San Diego County mental health system and include triage evaluators and case managers. Therapists complete a 4-hour training with 5 components: (1) intervention model and rationale, (2) safety procedures, (3) trial protocol, (4) fidelity monitoring procedures, and (5) mobile intervention deployment. Embedded in the training are role-plays of specific skills, including redirecting sessions to suicide-specific concerns and conducting risk assessments or documentation of adverse events. Therapists are required to complete role-plays concerning safety assessment, describing the treatment model, and re-establishing focus on suicide-related concerns in sessions.

### Randomization, Masking, and Trial Design Considerations

Participants are randomized to START with mobile augmentation or START alone. Randomization schedules are compiled by an independent statistician, and research assessors are kept masked to the assignment. Participants and the study therapists are not blinded to the assignment. Several factors led to our decision to include 2 active conditions in this pilot trial. First, we were encouraged by our prior trial that identified a statistically significant augmentative benefit of a mobile intervention immediately posttreatment with a comparable sample size, albeit in a somewhat different population. Second, we considered the primary success criteria for this pilot phase to start the absence of sustained changes in suicidal ideation (in aggregate) and secondary comparisons across the mobile-augmented and nonaugmented conditions were designed to yield information about preliminary differences in feasibility, acceptability, and hypothesized mediators of change (eg, recall of safety plans). Third, we considered a no-treatment control condition, but this design was not preferred by our stakeholder community partners. Moreover, due to the timing of the intervention to the gap period between urgent and ongoing care, a waitlist control would be impossible.

The primary success criteria for this pilot trial of START are sustained changes in suicidal ideation (in aggregate), and secondary comparisons across the mobile-augmented and nonaugmented conditions were designed to yield information about preliminary differences in feasibility, acceptability, outcomes, or hypothesized mediators of change (eg, outpatient engagement, recall of safety plans). Owing to the pilot nature of the trial, we may not have adequate power to detect modest differences between START and START with mobile augmentation conditions; however, we considered that the absence of detectable differences between conditions on any of the dimensions of feasibility, acceptability, mediators, and preliminary outcomes in the presence of sustained improvement in the primary outcome (suicidal ideation) would indicate that mobile augmentation may not be warranted in future deployment of START.

### Interventions

**Figure 1:**
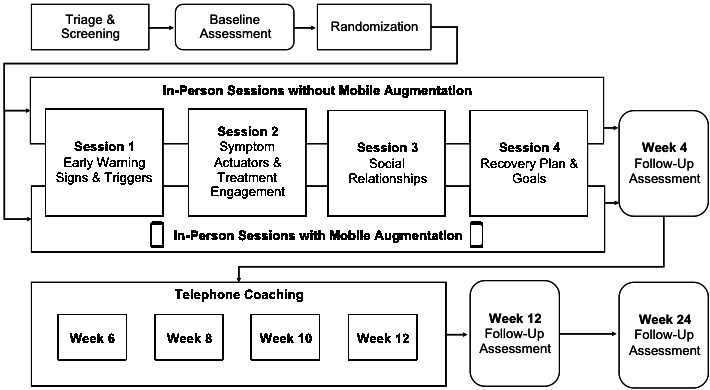
Study flow.

After baseline assessment, participants were scheduled for 4 consecutive weekly sessions with a study therapist in the walk-in facility. We opted for individual sessions (vs group) to enhance the likelihood of personalization and to reduce the potential for wait times to reach group capacity. Each session is structured with a demonstration and practice of a coping skill (10 min), skill focus on 1 of 4 topics (20 min), and personalization of implementation intentions surrounding a topic area (30 min). The structure of sessions is highly comparable between START with mobile augmentation and START-alone conditions, as both conditions involve compiling implementation intention statements in a workbook. Participants in the START-alone condition are instructed to record homework completion, and those in the mobile augmentation condition are instructed to respond to queries on the mobile app as described below. Figure 1 outlines the study flow.

#### Session 1: Content (Early Warning Signs and Triggers, 90 Min)

After establishing the ground rules, participants are led through a brief grounding technique called *look, point, name*. Then, an interactive description of the generic cognitive model is provided (ie, that thoughts, feelings, and behaviors are related), thoughts and feelings are identified and labeled, and patients complete exercises demonstrating how dysfunctional beliefs can impact feelings and behavior. Suicidal thoughts are discussed in this framework, and education is provided about the high frequency of such thoughts among people with SMI diagnoses. The concept of early warning signs (internal experiences) and triggers (external factors) are introduced in relation to suicidal thoughts. Participants are also presented with the idea of adaptive versus unhelpful responses; participants then form implementation intention statements based on adaptive responses. The session concludes with an agreement regarding the focus of work together in diminishing vulnerabilities to suicidal thinking, with parameters around safety planning, crisis contacts, and one commitment to reduce access to means. In the START with mobile augmentation condition, participants were provided with a 15- to 20-minute overview of interactive responding on the phone, and in the START-alone condition, the session concludes (see below for mobile content).

#### Session 2: Content (Symptom Actuators and Treatment Engagement, 60 Minutes)

In this session, the therapist first elects from 1 of the 2 symptom *actuators* of suicidal thoughts from early warning signs (mood or psychotic symptoms), with modules focused on these symptom clusters as they create vulnerabilities to suicidal thoughts. Evidence is used to challenge unhelpful beliefs, such as the uncontrollability or permanence of symptoms, and each is coupled with a behavioral suggestion or experiment that can be employed to test assumptions. The therapist and participant decide to focus on mood symptoms or voices, depending upon which is most associated with suicidal thinking. Participants delineate *portable* coping strategies for 3 levels of severity of depressed mood (and manic symptoms, if present) or for common dysfunctional beliefs about voices (eg, uncontrollability). In addition, the role of treatment in managing these symptoms is discussed, which is linked with intentional (eg, *treatment won’t work*) and unintentional (eg, forgetting) barriers and facilitators to treatment adherence.

#### Session 3: Content (Social Relationships, 60 Min)

This session begins with progressive muscle relaxation. Content then addresses the role of social relationships and beliefs about others as factors in coping with or exacerbating suicidal thinking. Unhelpful, generalized, or extreme beliefs about social interactions are reappraised (eg, *there are some people who are on my side*), and the role of social isolation in suicidal thinking is discussed. Potential behaviors that promote social contact are elicited, including the use of crisis-oriented resources. Barriers to asking for help are elicited, including appraisals and predictions about disclosing suicidal thoughts to others and behaviors that are linked with help-seeking that resulted in benefits.

#### Session 4: Content (Recovery Plan and Goals, 60 Min)

This session begins with a Loving Kindness meditation to increase positive affect and addresses recovery goals and future-oriented thinking; a recovery plan is developed around personal values and a linked single, attainable short-term goal consistent with personal values. Participants and the therapist worked within the START goal framework and delineated 2 relevant goals and steps aligned with the selected goals. Next, the therapist and participants reviewed the topics and content generated in sessions 1 to 4 and revised and added to statements that were previously generated. Plans for subsequent contacts were made for follow-up phone contacts.

#### Rationale for START Foci

The therapeutic targets were selected to address short-term risk and protective factors in a brief intervention format, and as such, do not directly address all of the risk factors for suicide in SMIs. For example, we do not directly target substance abuse, although participants do have the freedom to select substance use as a trigger or unhelpful coping strategy. We also do not directly involve caregivers, such as family members. We will use postintervention follow-up satisfaction data to identify potential modifications to the protocol.

#### Follow-Up Telephone Coaching

Participants are contacted via phone by the study therapist every at weeks 6, 8, and 10 or until they are established in outpatient care (ie, attend an intake appointment). Follow-up telephone coaching consists of a concise scripted interaction to briefly check in (target 10 min) with a focus on the following: (1) safety and utilization of strategies developed in the 4 in-person sessions; (2) assessing and problem solving around barriers to outpatient engagement; and (3) in the mobile augmentation condition, troubleshooting, and requests for adapting any elements of the device interaction [34-36].

### Mobile Augmentation Procedures

#### Mobile Devices and Training

Participants can opt to either use their mobile phone device or, if they do not use a personal mobile phone device, obtain a provisional one during the 12-week period with support for accessing free or low-cost mobile devices. In either case, the interactive content is delivered through an EMI app called Illumivu. Personalized content is delivered through this app, and deidentification is made possible by a mobile code that is unique to each participant. In Session 1, participants receive training that is individually tailored to their learning needs on how to respond to questions and response choices, procedures for charging the device, and responding to outreach.

#### Mobile Intervention Content

**Figure 2:**
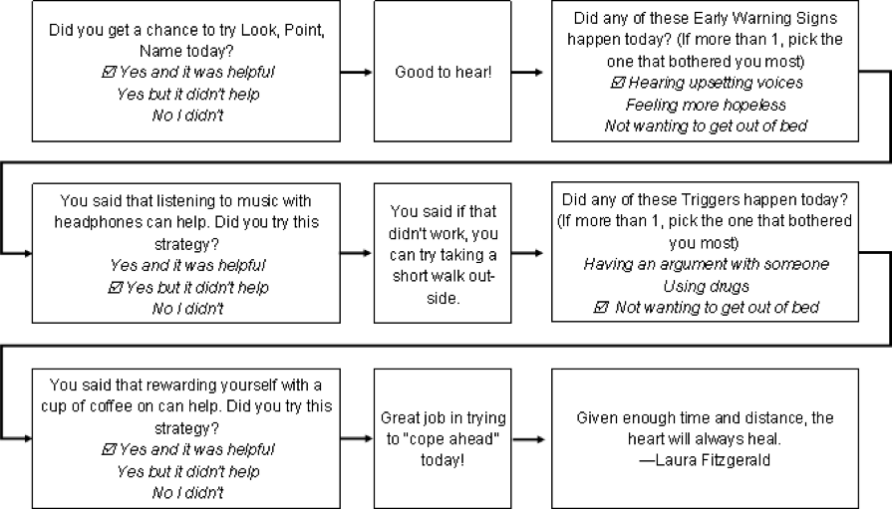
Sample mobile session for early warning signs and triggers.

Surveys scaffold onto in-person content and successively add modules according to the following schedule—*Session 1*: Early Warning/Triggers; *Session 2*: Symptom Actuators and Treatment Engagement; *Session 3*: Social Relationships; and *Session 4*: Recovery Plan/Goal tracking. In each survey, participants are asked whether they practiced relaxation skills. They are then asked to respond to *first-layer* ecological momentary assessment (EMA) questions that assess current state (eg, past 24-hour presence of triggers or early warning signs, severity of depressed mood). In the *second layer*, content is branched to address endorsement of maladaptive beliefs pertaining to first-layer questions, and the *third layer* contains 2 questions on participants’ adaptive cognitions or behaviors developed in session and their implementation intentions for these. Each of these layers contains a set of possible responses derived from content that was personalized in the in-person sessions. If no triggers or early warning signs or symptoms are endorsed, participants select from personalized adaptive beliefs and actions pertinent to the maintenance of wellness. Participants are then queried about their intention to engage in the behavior and the survey concludes with a quote on recovery, adapted from public sources or from anonymous prior participants. See Figure 2 for a sample mobile session. During in-person contact with study therapists, participants can request to alter content, add, remove, and/or update their responses. In addition to the *pushed* surveys, participants can access content (eg, recovery goals) on demand.

#### Rationale for Mobile Augmentation

Our group and others have completed trials that indicate that patients will use mobile devices for EMIs up to 12 weeks or more when coupled with in-person therapies [26,37]. On the basis of prior research suggesting that only 50% of participants initially engaged with outpatient care, there is reasonable concern that without additional intervention, participants may disengage from this care during the 6-month period of this study [38]. Given that there is no staff cost to continue an automated mobile intervention through 6 months after establishment in outpatient care and the content reinforces ongoing participation in care, the automated intervention is available in perpetuity.

### Safety Procedures

In collaboration with stakeholders, plans have been developed to systematically monitor and address safety. At *screening*, participants are seen on the same day as the triage assessment, in which plans for outpatient follow-up are set by the triage provider. Our research screening included crisis resources for all screened participants. If screening cannot occur on the same day as triage, we follow the protocol for baseline assessments. At baseline and follow-up assessments, we developed a suicide safety monitoring protocol that uses the CSSR-S to identify *increases* in ideation or new interim behavior since the time of screening (or in case of delay, urgent evaluation is initiated if CSSR-S scores are >2). During *in-person START sessions and telephone contacts*, the study therapist administers the CSSR-S at the end of each session. If an increase in ideation or new interim suicide behavior is identified, it activates urgent evaluation, connection with resources, and arrangement of notification of providers and hospitalization. We also elected not to directly query about suicidal ideation via the device, as the understanding of the use of remote technologies in querying suicidal risk and safety response is in its infancy [39,40].

### Measures

The measurement battery for this pilot trial was brief and restricted to study constructs related to acceptability and preliminary outcomes (Table 1).

**Table 1:** Study measures.

Timing and measure		Specific aim	Construct assessed
**Screening**			
	MINI^a^ DSM-5^b^ interview	Inclusion	Diagnosis
	CSSR-S^c^	Inclusion	Suicide risk inclusion
**Baseline and follow-ups**			
	Scale for Suicide Ideation or CSSR-S Interval	Primary outcome	Suicidal ideation severity
	Outpatient follow-up	Secondary outcome	Treatment engagement posttriage
	Composite suicide-related crises	Secondary outcome	Suicidal behavior or psychiatric hospitalization for suicidal ideation
	Beck Hopelessness Scale	Mechanism	Hopelessness
	Coping Self-Efficacy Scale	Mechanism	Self-efficacy
	EMA^d^ Adherence or Outcomes	Acceptability: secondary outcome	Response rate to mobile surveys: mood or psychotic symptoms
	Tablet routines questionnaire	Secondary outcome	Medication adherence
	BPRS-24^e^	Secondary outcome	Global psychopathology
	Treatment Rationale Scale	Acceptability	Treatment expectancy
	Timeline Followback Scale	Moderator	Substance abuse
**Follow-ups only**			
	Intervention Satisfaction Questionnaire	Acceptability	Satisfaction with intervention
	Recovery plan and safety plan recall	Mechanism	Accuracy of recall

^a^MINI: Mini International Neuropsychiatric Interview.

bDSM-V: Diagnostic and Statistical Manual of Mental Disorders Fifth Edition.

^c^CSSR-S: Columbia Suicide Severity Rating Scale.

^d^EMA: ecological momentary assessment.

^e^BPRS-24: Brief Psychiatric Rating Scale–24 item.

#### Suicidal Ideation Severity and Behavior (Primary Outcome)

We administer the interview-rated version of the *Scale for Suicide Ideation*, a 21-item widely used measure that predicts completed suicide [41,42] in addition to the interval CSSR-S, which queries the timing, severity, and characteristics of suicidal ideation and behavior [30].

Our study addresses changes in suicidal ideation and is underpowered to detect the impact on the risk of suicidal behaviors. Our rationale for change in suicidal ideation as our primary outcome was that for a suicide-specific intervention to be potentially effective, it must be feasible, acceptable, and associated with significant and sustained reductions in suicidal ideation to move on to a confirmatory trial. If the target of suicidal ideation was not moved, then we would not proceed to a confirmatory trial. We recognize that people may experience a reduction in suicidal ideation severity as part of natural regression to baseline. However, we note that people with SMIs are more likely to have chronic ideation than people without psychosis and therefore would be less likely to experience natural reductions in ideation [43]. Moreover, people with SMIs are more likely to have recurrent suicidal ideation, and as such, we added follow-up assessments at 12 and 24 weeks. Finally, we used the minimally important clinical difference of 0.5 SD as a threshold for meaningful reduction and power analyses. We will investigate whether the SSI score is skewed or kurtotic using the convention of +3 investigate skew and, if evident, will dichotomize the variable.

#### Outpatient and Crisis Service Utilization (Secondary Outcome)

We extracted electronic medical record (EMR) encounter data regarding attendance at the first follow-up appointment and rate of follow-up outpatient contacts. It is possible that participants may use out-of-county institutions not captured in the EMR; therefore, the *Cornell Service Use Index* [44] is administered for service use not recorded in the EMR to facilitate a comparison of augmented and nonaugmented arms.

#### Global Psychopathology (Secondary Outcome)

Global psychopathologic severity is evaluated using the *Brief Psychiatric Rating Scale–24-item expanded version 4.0* (BPRS-24) [45], a clinician-rated measure with 24 items that cover depression, anxiety, mania, suicidality, delusions or hallucinations, and unusual behavior.

#### Medication Adherence (Secondary Outcome)

Self-reported adherence is assessed with the *Tablet Routine Questionnaire* [46], which asks about the proportion of psychotropic medication taken over the past week and month.

#### Electronic Adherence and Targets (Secondary Outcome)

A wealth of EMA data will be generated, including adherence data (eg, response rate=the number of responses/number of queries) and patterns of adherence over time. In our completed trial, treatment response was associated with greater adherence; thus, we will also explore this association. We structured the EMA protocol to provide day-to-day data on the early warning signs or triggers, symptoms, medication use, socialization, and contextual influences. These data will inform hypotheses about determinants or contexts of suicidal thoughts in SMIs [47].

#### Substance Use Frequency or Intensity (Moderator)

We assessed substance abuse as an exploratory moderator of attrition, adherence, and response. To quantify alcohol and drug use, we selected the 30-day *Timeline Followback Scale* [48].

#### Therapeutic Mechanisms Variables

We examined mechanistic targets for the START intervention that map on to therapeutic content: *Treatment Engagement*, *Coping Self-Efficacy (CSE) Scale* [49] and *Beck Hopelessness Scale* [50].

#### Safety and Recovery Plan Recall (4, 12, and 24 weeks only)

Modeled after recent research on recall of elements of CBT for bipolar disorder [51] and our research in schizophrenia [15], we administer a knowledge or recall measure that corresponds to elements in the safety plan generated in Session 1 as well as the recovery plan in Session 4. Participants are asked to recall broad elements discussed (eg, responding to early warning signs) and the individual responses of their own plan once prompted (eg, specific responses to early warning signs). Participants’ written responses are coded as fully accurate, partially accurate, or not accurate in regard to recalled elements of the safety and recovery plans.

#### Acceptability Measures

At baseline, participants are administered the *Treatment Rationale Scale* [52], a 3-item self-report that garners perceived credibility and anticipated benefit. Participants also completed seven 5-point Likert-type and 4 open-ended questions (modified from Kimhy et al [53] and used in our prior research [54]) focused on greater intervention satisfaction, barriers and suggestions concerning the intervention, the therapist, and the manual, and for the mobile health condition, experiences with the device and suggestions for future usability.

#### Fidelity

We systematically address the relevant components of treatment integrity: *Competence, Therapist and Participant Adherence, and Treatment Differentiation* [55]. *Competence* is addressed by initial training of the study therapists who meet minimum competence standards (see above) and in an ongoing fashion by audiotape-rated delivery of the intervention, supported by weekly supervision. *Therapist Fidelity* to the manualized protocol is assessed via 100% of session recordings rated by the therapist and research team on an adapted version of the Cognitive Therapy Rating Scale for Psychosis (CTS-Psy) [56]. *Participant adherence* is assessed by session attendance or telephone contacts and objectively via device-obtained data. *Treatment differentiation* is assessed by blinded random selection of 25% of audio-taped sessions rated by masked raters, with a running quarterly calculation of discrepancies between conditions evaluated to determine if content delivered varies between the 2 conditions; retraining occurs if differences in CTS-Psy scores are >0.5 SD.

#### Sample Size Determination

Our pilot trial’s sample size is derived based on the nexus of 3 goals, guided by work on optimization of pilot studies [57]: (1) to obtain point estimates of feasibility, acceptability, and test the impact of the intervention compared with population base rates for engagement and crisis service utilization; (2) to be powered to test a clinically significant within-subjects reduction (*d*>0.5) in the SSI score; and (3) to evaluate the preliminary impact of augmentation. Intent-to-treat analyses with 70 patients, with an alpha of .05, will have 95% power to evaluate whether the sample estimates differ from population estimates (eg, 50% no-show rates) [58] and 77% power for each of the individual arms. On the basis of the General Linear Multivariate Model Power and Sample Size program [59], there is >0.80 power across 24 weeks to detect a 0.5 SD pre-post change in SSI score. We have considerably less power to detect augmentation effects, but following recent research on sample size estimation in pilot trials and confidence intervals [60,61], the preliminary impact of augmentation will be assessed by defining a minimally clinically significant augmentative effect for a future trial.

#### Analysis of Feasibility and Acceptability

Point estimates of feasibility and acceptability included the following: (1) triage clients screened, (2) screened clients eligible, (3) eligible clients enrolled, (4) enrolled clients completing 4 sessions and follow-up calls, and (5) percentage of participants reporting being somewhat or very satisfied with the overall intervention. Comparative data regarding feasibility and acceptability are our completed trials, which enable us to examine whether indicators of feasibility in the triage setting are similar to general outpatient populations. Each of these estimates, in addition to Treatment Rationale and Satisfaction scores, will be contrasted across conditions with *t* tests or Mann-Whitney tests or chi-square tests.

#### Analysis of Within-Person Changes

Distributions for the SSI score may be skewed (with overdispersion due to persons with no present ideation) and, if so, zero-inflated negative binomial models will be employed. We will use hierarchical linear models (HLMs) in which time will be tested as a categorical time-varying predictor of SSI score and subjects are a random effect, with significant reduction in SSI over time indicated by a statistically significant time effect. We expect that by the 24-week time-point, SSI will have at least a medium effect size decrease (*d*≥0.5) as measured by HLM linear-estimated change. To evaluate augmentation, we will examine group×time interactions, with estimated standardized differences considered supportive of the augmentation inclusive of minimal clinical significance CI>0.80. We will evaluate secondary continuous outcomes (eg, BPRS) variables in the same way.

#### Analysis of Mechanisms

For analysis of binary mechanisms (outpatient engagement) and outcomes (crisis service use), we will use chi-square analyses to examine associations at 24 weeks. For the continuous time-varying mechanism (CSE), we will examine a mechanistic association between changes in CSE and SSI scores using the Macarthur framework [61]. We will use generalized estimating equations to evaluate the prediction of crisis service use by within-subjects change in CSE and then repeat these analyses within mobile-augmented and nonaugmented arms. We will also contrast the Safety or Recovery Plan recall at weeks 4, 12, and 24 using *t* tests or Mann-Whitney U tests.

## Results

The trial is ongoing and recruitment is active, with anticipated completion of the baseline sample randomization target of 70 participants by the close of 2020.

## Discussion

### Principal Findings

This trial focuses on evaluating a novel brief suicide prevention intervention for SMI, addressing the imbalance between the low number of empirically supported interventions for SMI and the high rates of suicide in people with bipolar disorder and psychotic disorders. Key considerations in developing and deploying an intervention to fit the needs of people with SMI were in the design of the intervention content and the setting and timing of the intervention within the typical American community mental health care continuum. This approach could yield potential benefits to both suicide prevention and operational efficiency by improving follow-through with scheduled outpatient appointments and reducing lost productivity due to *no shows* at follow-up appointments. This experimental treatment approach is an augmentation of the standard transition from a walk-in clinic to outpatient care follow-up and is not fully integrated into care. The likelihood of future adoption may depend upon a demonstrated reduction of suicide and cost recovery due to the reduced rate of missed appointments.

A trial focus is also on the relative additional value of integrating mobile interventions with in-person appointments. The additional mobile intervention content is meant to augment the recall and implementation of personalized intervention content derived from in-person sessions. In this community-based trial of a suicide-specific intervention, we did not have a no-treatment control condition, and this trial design decision was due in part to the community partner’s preference against a no-treatment condition. We note that future developmental trials of suicide-specific interventions may face a similar dilemma and alternative designs (eg, SMART designs) may provide useful means of detecting mobile augmentation effects.

### Lessons Learned

Key lessons learned to date include the importance of frequent deployment meetings that emphasized co-design among staff training and leadership to enable referral, recruitment, and safety protocol refinement. We also based our safety protocol on published guides [29]; these guides have yet to be specifically adapted for SMIs but provide a reasonable starting point for clinical trials in suicide prevention in SMIs. We also opted not to include direct questions about suicidal ideation or behavior during automated mobile communications that augment in-person appointments, due to the lack of specific evidence of acceptability in SMI to date. The trial is currently ongoing, and we anticipate that data collected will further inform research and practice in suicide prevention interventions adapted for SMIs.

## Abbreviations

BPRS-24: Brief Psychiatric Rating Scale–24 item
CBT: cognitive behavioral therapy
CSE: coping self-efficacy
CSSR-S: Columbia Suicide Severity Rating Scale
CTS-Psy: cognitive therapy rating scale for psychosis
EMA: ecological momentary assessment
EMI: ecological momentary interventions
EMR: electronic medical record
HLM: hierarchical linear model
SMI: serious mental illnesses
SSI: Scale for Suicide Ideation
START: SafeTy and Recovery Therapy
